# Reliability and validity of the Greek translation of the patient assessment of chronic illness care + (PACIC-PLUS GR) survey

**DOI:** 10.1186/s12875-020-01192-z

**Published:** 2020-06-25

**Authors:** Maria Malliarou, Eleni Bakola, Athanasios Nikolentzos, Pavlos Sarafis

**Affiliations:** 1 Department of Nursing, Perifreiakh Odos Larisas –Trikalon, University of Thessaly, 41110 Larisa, Greece; 2 The General Panarkadiko Hospital of Tripoli “I Evagelistria”, Terma Erithrou Stavrou, 22100 Tripoli, Greece; 3 Hellenic Open University, Parodos Aristotelous 18, 26335 Patras, Greece; 4 Department of Nursing, Cyprus University of Technology, 30 Archbishop Street, 3036 Limassol, Cyprus

**Keywords:** Instrument evaluation, Chronic patients, Chronic care model, PACIC+

## Abstract

**Background:**

This study aimed to investigate the Patient Assessment of Chronic Illness Care+ (PACIC+) which is a tool to assess care for Chronic Conditions combining PACIC items with an overall 5As score derived from the ‘5As’ model (ask, advise, agree, assist, and arrange), and is congruent with the Chronic Care Model. In addition, the study at hand aimed to translate the PACIC+ tool into Greek and test its psychometric properties to the Greek patients.

**Methods:**

Questionnaires were collected from 268 chronic patients. Internal consistency and reliability were determined by the calculation of Cronbach’s alpha coefficient. A confirmatory factor analysis (CFA) was conducted in order to test the construct validity of the questionnaire. Validity was further examined by investigating the correlation of PACIC+ with SF-36 and its association with sex and age.

**Results:**

Internal consistency reliability was accepted with a Cronbach’s alpha above 0.70 for all PACIC+ dimensions. CFA showed that the 10-dimensional model fitted the data well (RMSEA = 0.059, CFI = 0.91 and GFI = 0.83). Most of the correlations coefficients between PACIC+ and SF-36 dimensions were significant. A significant and negative correlation was found between PACIC+ summary score, Patients’ activation and Goal Setting/ Tailoring with age.

**Conclusions:**

The Greek translation of the PACIC+ questionnaire has good psychometric properties and has proven to be a credible and valid tool to be used by Greek researchers in order to measure patients’ perceived care during treatment. It demonstrated high reliability and internal consistency, extending the applicability of this instrument to Greek speaking chronic patients.

## Background

The pressure exerted by the growing demand for long term care of chronic patients on the health system is annually increasing [1]. The prevalence of chronic illness is constantly increasing and is largely due to demographic aging and a multitude of socio-economic risk factors [2]. The Chronic Care Model characterizes chronic illness as a condition requiring constant interaction between the patient and the health care system [3].

The scarcity of available financial resources for health, the increased expectations and the diversification of the population’s needs have led policy makers in finding ways and tools of assessing the effectiveness of medical and thus achieving a more rational way of distributing health care recourse [4]. A patient-centered care has been considered necessary in order to assess the needs and the preferences of chronic patients as the aforementioned care takes into account patients’ preferences and values, provides psycho-physiological comfort, highlights the importance of communication and the need to provide support and coordinated care to the patient, and is the key in general in improving health services. In addition, the involvement of administrative, medical and nursing leadership is considered necessary in the management of chronic care [5]. Proper management of chronic illness has been proven to reduce the consequences of non-adherence with medication regimens worldwide. Managing chronic illness, and especially when more than one coexists, requires a transformation in the health system. Almost half of the chronically ill patients suffer from more than one pathological condition therefore, a more comprehensive approach is required. The healthcare system has shown particular interest in the past, to integrate or correct management deficiencies of current models and systems of management of chronic illnesses such as diabetes, cardiovascular diseases, asthma and hypertension [6–8]. Therefore, integrated healthcare systems have shown an early interest in correcting shortcomings in the management of chronic illness [8–11].

Addressing these shortcomings requires a transformation of the healthcare system, from one that is tedious and responds only to morbidity, to a dynamic active system that focuses on maintaining a person’s health, on preventing relapses and on achieving proper management of chronic illness [9, 12, 13]. In order to accelerate such a transition in 1998, the Improving Chronic Illness Care program created the Chronic Care Model (CCM) for healthcare systems. After the implementation of the CCM several studies have produced data on its effectiveness [14].

In Greece, there are no studies regarding the assessment of the care provided to patients with a specific patient-centered measurement tool. This study aimed to translate the PACIC+ tool from English into Greek and to test its psychometric properties, and therefore enabling the assessment of the different aspects of the CCM and the 5A model for Greek patients. This procedure is crucial for indicating a proper instrument for accessing health care provided to chronically ill patients in Greece and therefore for using it any future research projects. For this purpose, we employed the use of “Patient Assessment of Chronic Illness (PACIC+)” tool [15], which was translated in Greek, on chronic patients who were hospitalized in a Public General hospital in order to test its validity and reliability.

In addition several correlations with socio-demographic characteristics such as gender, age etc. with PACIC+ were conducted, since gender and age differences are rather common to be investigated and quality of life levels may vary according to different sexes and age.

Validating scales such as the PACIC+ is a rather intriguing and complex task. Although convergent validity with certain related scales (for example alternative patient self-report measures of quality) is rather useful [16, 17], construct validity is complicated due to the fact that socioeconomic and demographic variables such as sex, gender, economic status and multimorbidity do not relate in a clear and profound way to PACIC+ scores. International studies have presented predicted relationships with measures of self management behavior [18, 19] and self-rated health [20, 21].

The Chronic Care Model, as it was already mentioned, identifies the key elements of a health care system that encourage high quality care for chronic illness. It focuses on provision of care to chronically ill patients either in the community (community care) or in health care organizations. The specific characteristic which the CCM intends to access are the following: giving support to chronically ill patients for self-management, promoting interaction with patients, integrating guidelines with patient preferences, coordinating healthcare teams promoting regular interaction between caregivers and patients [16]. In addition, it introduces substantial concepts of change to each element and combines the promotion of productive interactions between well-informed patients who are actively taking part in the care of their illness. It also introduces resources along with the expertise of the providers [16, 22, 23]. The absence of an instrument that could measure the patient assessment of care for chronic conditions, from the patient side, led Glasgow et al. (2005) to create the “Patient Assessment of Care for Chronic Conditions (PACIC)” tool according to the CCM model [15, 23–25]. The tool has been translated and tested in several European countries and has been used with patients with different chronic diseases such as diabetes, hypertension, cardiovascular diseases and asthma [19, 26–34]. The PACIC tool was initially tested and validated in a population of mostly white, English-speaking patients with various chronic illnesses, followed by another validation for the Hispanic/Latino population in the United States of America [35] The Patient Assessment Chronic Illness Care (PACIC) questionnaire is a patient reported instrument which assesses quality of patient-centred care for chronic illness in consistency with the CCM. The PACIC+ contains the exact 20 items as the PACIC, with the inclusion of six items. The items were selected from the so-called ‘5As’ model (ask, advise, agree, assist, and arrange), which essentially is a patient driven model dedicated to behavioural counselling. Its distinctive feature is that it is in accordance to the basic principles of the CCM and that it has been often deployed to encourage self-management maintenance and affiliations to community resources. The PACIC+ integrates the aforementioned with existing PACIC items, hence allowing scoring of five-item subscales on delivery of each of the ‘5As’, in addition to an overall 5As score [36].

In relation to the details of the PACIC+ questionnaire it is worth mentioning that it consists of 26 items, with a 5-point rating scale anchored from 1 (Almost never) to 5 (Almost always). Each subscale is scored by averaging the answers of each item in the subscale. Subscales take values between 1 (Almost never) and 5 (Almost always). Higher rankings show healthcare provision in harmony with the CCM and chronic patients being actively involved in self-management of their disease, while lower rankings indicate that health care is not harmonized with CCM. The first 20 items of the questionnaire fall into one of five subscales presented in the following order: Patient Activation (1–3 items), Delivery System Design/Decision Support (4–6 items), Goal Setting (7–11 items), Problem-solving/Contextual Counseling (12–15 items) and Follow-up/Coordination (16–20 items). In addition the next 6 items derive from the patient-centered model of behavioral counseling “5As”. The combination of the 6 items with the existing PACIC 20 items, allows the scoring of five-item subscales on delivery of each of the “5As” (ask, advise, agree, assist, arrange), as well as an overall 5As score [37]. Finally, the PACIC+ tool succeeds in addressing the evidence-based 5A model for behavioural changes [38].

Both questionnaires, PACIC and PACIC+, were subjected to factor analysis by Glasgow et al., (2005) and by Glasgow, Whitesides, Nelson and King (2005) respectively [16, 19].

## Methods

### Participants and procedure

The survey was conducted at a General Public Hospital in Western Greece and its participants were patients who were admitted to the hospital due to their chronic disease and not because of any acute reason (surgery, trauma etc). The study did not include any patients from the Intensive Care Unit, the Operating room and the Emergency Department due to their inability to participate in completing the questionnaires. A written informed consent was obtained from each participant and the self-completed questionnaires were collected in January, February and March 2017. In order to calculate the correct sample size for the study the researchers followed Hatcher’s and O’Rourke’s [39] recommendations for a minimum sample size of 5 times the number of variables aka 100 subjects. In the study at hand the questionnaire included 26 items, which indicated that a minimum requirement of 130 participants was necessary. However, an additional drop-out rate of 15% was taken into account and as a result a target of at least 150 participants for the study was decided.

In order to use the translated into Greek version of PACIC+ in our study a written permission was granted by the MacColl Center for Health Care Innovation. The first two translations in Greek from the original English text were conducted independently by 2 nursing Professors. Subsequently the reverse translation was then conducted by a native English speaker. Two experts of the nursing scientific community compared the two manuscripts and gave positive feedback enabling our research team to implement their constructive comments. In terms of reliability, internal consistency was assessed at both scale and subscale level. As far as convergent validity is concerned, floor and ceiling effects, factor structure, and associations between the PACIC+ and the quality of life dimensions included in the SF-36 questionnaire were explored. SF-36 was chosen because it is a generic instrument of measuring health related quality of life and it is appropriate for such explorations.

### Data analysis

Continuous variables are presented with mean and standard deviation (SD) or with median and interquartile range (IQR). Quantitative variables are presented with absolute and relative frequencies. A confirmatory factor analysis (CFA) with maximum likelihood procedure was conducted in order to test how well the PACIC model fits the data. The variance of the latent constructs was fixed at one during parameter estimation and the factors were allowed to be correlated. The fit of the CFA model was assessed using the comparative fit index (CFI), the goodness of fit index (GFI), the standardized root mean square residual (SRMR) and the root mean square error of approximation (RMSEA) [34]. For the CFI and GFI indices, values close to or greater than 0.95 are taken to reflect a good fit to the data [37]. SRMR values of 0.08 or less are being indicative of an acceptable model, while RMSEA values of less than 0.05 indicate a good fit and values as high as 0.08 indicate a reasonable fit [40, 41]. Exploratory factor analysis (EFA) was also carried out to evaluate construct validity and to disclose any underlying structures of the study questionnaire. Principal component analysis (PCA) was chosen as an extraction method using Varimax rotation. The cut-off point for factor loadings was set to 0.40 and for Eigen values it was 1.00.

Scale internal consistency was determined by the calculation of Cronbach’s α coefficient. Scales with Cronbach’s α coefficient equal to or greater than 0.70 were considered acceptable. Polychoric correlations were used to explore the association of PACIC+ items [41]. Validity was further examined with the correlations (Pearson’s r) of PACIC+ total score with the SF-36 dimensions. Differences on PACIC+ total score between men and women were evaluated by the use of Student’s t-test. Pearson’s correlation coefficient was used to explore the association of PACIC+ total score with age. *P* values reported are two-tailed. Statistical significant level was set at .05 and analysis was conducted using SPSS, AMOS (SPSS, Chicago, IL, USA) and STATA Statistical Software.

## Results

Data from 268 participants (122 men and 146 women) were collected and analysed. Sample characteristics are shown in Table 1. Total sample mean age was 61.4 (*SD* = 14.4). Most of the participants were married (66.4%) and the majority was Greek (94.0%). Almost half of the participants were retired (52.6%) and 77.2% had a public insurance coverage.

**Table 1:** Sample characteristics

	N (%)
**Gender**	
Men	122 (45.5)
Women	146 (54.5)
**Age (years),*M*(*SD*)**	61.4 (14.4)
**Ethnicity**	
Greek	252 (94.0)
Other	16 (6.0)
**Family status**	
Single	26 (9.7)
Married	178 (66.4)
Divorced	20 (7.5)
Widowed	44 (16.4)
**Educational years**	
≤ 6	125 (46.6)
7–12	84 (31.3)
> 12	59 (22.0)
**Working status**	
Unemployed	40 (14.9)
Employed	87 (32.5)
Retired	141 (52.6)
**Insurance**	
Private	34 (12.7)
Public	207 (77.2)
Uninsured	27 (10.1)

Our analysis on floor and ceiling effects of the PACIC+ items indicated that floor effects ranged from 3% (item 5) to 31.3% (item 16), while ceiling effects ranged from 3,7% (item 17) to 27.6% (item 5). Most of the responses were spread among the possible answers. Polychoric correlation of the PACIC+ items ranged from 0.3 to 0.74 and the mean polychoric correlation coefficient was equal to 0.43, indicating that the association between the items was significant.

Mean values and Cronbach’s alpha coefficients for PACIC+ scales are presented in Table 2. All the scales of PACIC+, exceeded the minimum internal consistency standard of 0.70 and ranged from 0.71 (Delivery System/Practice Design) to 0.83 (Problem Solving/ Contextual). Mean summary score was 2.9 (*SD* = 0.8) and the Cronbach’s alpha for total PACIC+ was .93.

**Table 2:** Descriptive Statistics of PACIC+ Subscales and Cronbach’s Alpha Coefficients

	Minimum	Maximum	M	SD	Cronbach’s a
PACIC+ summary score	1.0	5.0	2.9	0.8	.93
Patient activation	1.0	5.0	2.9	1.0	.79
Delivery System/PracticeDesign	1.0	5.0	3.3	0.9	.71
Goal Setting/ Tailoring	1.0	5.0	2.8	0.8	.80
Problem Solving/ Contextual	1.0	5.0	3.2	0.9	.83
Follow-up/ Coordination	1.0	5.0	2.6	0.9	.81

As defined from the CFA results, the 5-dimensional model fitted the data since the fit indices results of the model did not reach the expected values and no strong evidence of the hypothesized model of Glasgow et al. was found. The *RMSEA*, SRMR, *CFI* and *GFI* values were 0.059, 0.09, 0.91 and 0.83, respectively. None of the item cross loadings exceeded the item loadings on the intended latent construct. Factors loadings were high and ranged from .62 to .83. Correlation between the PACIC+ factors is shown in Table 3 and was high indicating the existence of a simpler factor. Thus, a CFA examining the unidimensionality of the PACIC+ was conducted and the fit indices were improved. The *RMSEA*, SRMR *CFI* and *GFI* values were 0.048, 0.072, 0.94 and 0.91, respectively revealing the existence of a single factor.

**Table 3:** Correlations between the PACIC+ factors

	Patient activation	Delivery System/Practice Design	Goal Setting/ Tailoring	Problem Solving/ Contextual
Delivery System/Practice Design	.65			
Goal Setting/ Tailoring	.64	.66		
Problem Solving/ Contextual	.64	.64	.76	
Follow-up/ Coordination	.58	.71	.67	.60

An exploratory factor analysis with principal component method and with varimax rotation was conducted on the sample. Using the latent root criterion of retaining factors with Eigen values greater than 1.0, a three-factor structure was identified, with the extracted factors explaining 58% of the total variance. Kaiser-Meyer-Olkin Measure of Sampling Adequacy was equal to 0.91 indicating that the PACIC+ items were suitable for factor analysis. Factor loadings (over 0.4) ranged from 0.44 to 0.80. Fifteen of the items loaded into one factor (1–15), while the other two factors had two (items 16, 17) and three items (items 18, 19, 20), respectively. The existence of one factor with most of the items and also the existence of secondary loadings of the items 1, 2, 7, 9, 14, 16, 17 and 20 further indicated that a single factor may have been more suitable for the data.

Correlation coefficients between PACIC+ total score and SF-36 dimensions are presented at Table 4. A significant and positive correlation between the PACIC+ score with most of the SF-36 dimensions was found.

**Table 4:** Correlations of PACIC+ with SF-36 Dimensions

	PACIC –plus Summary score
Physical Functioning	.19**
Role-Physical	.13*
Bodily Pain	.12
General Health	.22***
Vitality	.09
Social Functioning	.28***
Role-Emotional	.02
Mental Health	.16*
Physical Component Summary	.21**
Mental Component Summary	.11

*Note.* *p < .05. p** < .01. ***p < .001

No significant differences were found in PACIC+ score between men and women. A significant and negative correlation was found between PACIC+ summary score with age (r = −.14, *p* < .05).

## Discussion

All subscales of PACIC+, exceeded the minimum reliability standard of 0.70 and ranged from 0.71 (Delivery System/Practice Design) to 0.83 (Problem Solving/ Contextual) and they were comparable to other research studies carried out in order to study translated versions of PACIC+, such as the Spanish version [42] or the Dutch version [43]. Mean summary score was 2.9 (*SD* = 0.8) and the Cronbach’s alpha for total PACIC+ was .93. This research found no significant differences in PACIC+ subscales scores between men and women, however, a significant and negative correlation was found between PACIC+ summary score, Patient activation and Goal Setting/ Tailoring with age. Research studies on demographic correlations with PACIC+ have not reached to an ultimate or definite conclusion, since several studies suggest that correlations may be observed, though other studies reach opposite conclusions. For example, Glasgow, Whitesides, Nelson and King (2005), point out that PACIC+ scores should not be related to patients’ demographics as their results are confusing. This means that sometimes they seem to correlate significantly and sometimes not [19]. However, while Glasgow and colleagues were not able to demonstrate significant differences in PACIC+ scores regarding patients’ socio-demographic characteristics within their particular research setting in the United States of America [16, 19], Rosemann et al. managed to identify significant differences on the basis of age, education and psychiatric symptoms for patients who received health care in specific European health care settings [44]. Hence, the latter findings are of important value as it is suggested that the fact that all patients’ groups may benefit to the same extent from advances in chronic illness care is a crucial factor in the implementation of the CCM.

The convergent validity analysis also indicated that the PACIC+ model showed a reasonable pattern of associations. The factorial structure of the PACIC-plus questionnaire is capable of revealing patient understanding through their own involvement, beliefs and concerns about their healthcare. It allows the general assessment of the patients, the perception and the satisfaction they receive from the health care. Finally, it also permits patients to interpret their awareness of their role as consumers taking care of their health services they receive and assessing their quality of life.

With respect to the relationships between the generic PACIC+ dimensions and the SF-36 scales, it can be argued that correlations between the two instruments were as predicted. The SF-36 Health Survey questionnaire has been widely used in recent years to assess the quality of life and has been the validated and translated into Greek by Pappa, Kontodimopoulos and Niakas (2005) [45]. The most significant correlations emerged between scales and dimensions tapping similar aspects such as patient activation with physical function and problem solving/contextual with general health and social functioning. The positive correlations between several dimensions of care and quality of life showed that better care of patients resulted in an improved quality of life. This comes in agreement with previous studies that came to the conclusion that patients with chronic conditions generally experience lower Quality of Life and subsequently several aspects of their life may be negatively affected [46].

Given the increase in the prevalence of chronic illnesses in Greece [47], improving people’s health with chronic conditions has become a priority for patients, healthcare providers, insurance providers and policy makers. The use of CCM and the PACIC+ questionnaire is proposed to guide change in hospital and community environments [48–50] and to reform health systems, thus making it imperative for practical and validated assessment tools.

Our research limitation was that the population studied derived from a single healthcare service. The survey’s sample was heterogeneous with patients presenting varied characteristics and suffering from a variety of diseases. Because the understanding and awareness of the principles of the CCM is extremely variable and influenced by both the uniqueness of the patient’s factors, the care and the models that are already in place, it would be desirable to define patient understanding of concepts as well as the care model used in future research. Another research limitation might be the use of QoL instrument for validation. However, quality of integrated care or self-management instruments were not suitable to be used as they are not psychometrically tested.

## Conclusions

In summary, the Greek PACIC+ has good psychometric properties and has proven to be a credible and valid tool to be used by Greek researchers in order to measure patients’ perceived care during treatment. The factorial composition of the PACIC+ continues to be consistent with the underlying theoretical framework and the related literature. Furthermore, it demonstrated high reliability and internal consistency, thus indicating a high level of applicability to Greek speaking chronic patients. The Greek version of PACIC+ questionnaire can be a useful patient centered instrument.

## Data Availability

The datasets generated and analyzed during the current study are not publicly available to preserve the privacy of the participants but are available from the corresponding author on reasonable request.

## Abbreviations

PACIC+: Patient Assessment of Chronic Illness Care plus
CCM: The Chronic Care Model
SF-36: The Short Form (36) Health Survey
CFA: Confirmatory Factor Analysis
IQR: Median and Interquartile Range
CFI: Comparative fit index
GFI: Goodness of fit index
RMSEA: Root mean square error of approximation
SD: Standard Deviation
SPSS: Statistical Package for the Social Sciences
AMOS: A statistical software package for structural equation modelling, produced by SPSS
